# A Magnetic-Coupled Nonlinear Electromagnetic Generator with Both Wideband and High-Power Performance

**DOI:** 10.3390/mi12080912

**Published:** 2021-07-30

**Authors:** Manjuan Huang, Yunfei Li, Xiaowei Feng, Tianyi Tang, Huicong Liu, Tao Chen, Lining Sun

**Affiliations:** 1 Jiangsu Provincial Key Laboratory of Advanced Robotics, School of Mechanical and Electric Engineering, Soochow University, Suzhou 215123, China; szdxhmj@126.com (M.H.); szdxfxw1014@163.com (X.F.); 20195229029@stu.suda.edu.cn (T.T.); chent@suda.edu.cn (T.C.); lnsun@hit.edu.cn (L.S.); 2 Harbin Institute of Technology, School of Mechatronics Engineering, Harbin 215123, China; liyunfei3321@foxmail.com

**Keywords:** vibration energy harvesting, electromagnetic generator (EMG), nonlinear, magnetic coupling, wideband, high performance

## Abstract

This paper proposed a high-performance magnetic-coupled nonlinear electromagnetic generator (MNL-EMG). A high-permeability iron core is incorporated to the coil. The strong coupling between the iron core and the vibrating magnets lead to significantly improved output power and a broadened operating bandwidth. The magnetic force of the iron core to the permanent magnets and the magnetic flux density inside the iron core are simulated, and the dimension parameters of the MNL-EMG are optimized. Under acceleration of 1.5 g, the MNL-EMG can maintain high output performance in a wide frequency range of 17~30 Hz, which is 4.3 times wider than that of linear electromagnetic generator (EMG) without an iron core. The maximum output power of MNL-EMG reaches 174 mW under the optimal load of 35 Ω, which is higher than those of most vibration generators with frequency less than 30 Hz. The maximum 360 parallel-connected LEDs were successfully lit by the prototype. Moreover, the prototype has an excellent charging performance such that a 1.2 V, 900 mAh Ni-MH battery was charged from 0.95 V to 0.98 V in 240 s. Both the simulation and experiments verify that the proposed bistable EMG device based on magnetic coupling has advantages of wide operating bandwidth and high output power, which could be sufficient to power micro electronic devices.

## 1. Introduction

With the maturity of low-power wireless transmission technology, various types of micro sensors, embedded systems, and wireless sensor networks have been developed rapidly [1]. The power consumption of micro electronic devices has been reduced to the order of microwatts. Due to the short service life, limited stored energy and environmental pollution problems, traditional chemical batteries are not suitable for the power supply of microelectronic devices with complex application environments [2,3,4].

Vibration energy harvesting technology converts vibration energy into electrical energy through electromechanical conversion mechanisms such as electromagnetic [5,6,7,8], piezoelectric [9,10,11,12,13], electrostatic [14,15,16], and triboelectric [17,18,19,20]. Among them, piezoelectric generators (PEGs) are especially attractive due to their simple construction, compact size, high power density and easy manufacturability, and PEGs are widely used in low-power electronic devices such as embedded systems and wireless sensing network nodes. Kim et al. [21] developed a flexible P(VDF-TrEE) film-based PEG on PDMS substrate with high power density, and the demonstrated output voltage, current, and power density were 5.8 V, 3.2 μA, and 6.62 mW/cm^3^, respectively. Triboelectric nanogenerators (TENG), on the other hand, have attracted extensive attention and research because of its high output voltage, simple fabrication, low cost, and wide application of materials [22]. Han et al. [23] presented an r-shaped hybrid piezo/triboelectric nanogenerator to enhance the output performance. With a 5 Hz periodic external force, the output voltage, current, and power density of the triboelectric part were 240 V, 27.2 μA, and 2.04 mW/cm^3^. Ma et al. [24] designed and investigated an integrated electromagnetic-triboelectric-piezoelectric hybrid generator. The tested short-circuit currents of electromagnetic generator (EMG), TENG, and PEG are 21 mA, 4.1 μA, and 0.7 μA. Furthermore, the maximum instantaneous output powers of EMG, TENG, and PEG are 30.9 mW, 712.3 μW, and 6.37 μW, respectively, under the respective external load resistances of 200 Ω, 70 MΩ, and 50 MΩ. Therefore, compared with PEG and TENG, EMG has the advantages of low impedance and high current output, and the output power can meet special requirements.

At present, most of the vibration generators are linear designs, which can achieve the maximum output power only when the resonance is consistent with the external vibration source [25]. Once the frequency of the vibration source deviates from the resonance of the generator, the output power will decrease rapidly, indicating a relatively narrow working bandwidth [26]. In general, the environmental vibration sources are random and cover a certain range, the working frequency of the vibration generators need to be broadened to achieve high output performance. To increase the output power and broaden the operating bandwidth of the vibration generators, various approaches, such as oscillator arrays [27], multi-modal oscillators [28,29], and active or passive frequency tuning technologies [30] have been proposed. In addition, researchers have exploited numerous approaches to introduce nonlinearity into vibration generators, including monostable Duffing oscillators [31,32], bistable oscillators [33,34,35], and frequency-up-conversion technologies [36,37] etc. The above mechanisms are commonly used in PEGs, EMGs, and TENGs. Stanton et al. [38] designed a bistable broadband PEG by adding a pair of repulsive magnets to a PZT cantilever. Bouhedma et al. [39] proposed a dual-frequency PEG with integrated magnets in a folded beam resonant structure. A bidirectional frequency tuning is achieved by adjusting the position of the magnet, which greatly broadens the operating bandwidth of the system. Magnetic spring is the most investigated structure among nonlinear EMGs. A traditional magnetic spring-based generator consists of two (top and bottom) fixed magnets with a third magnet levitated between them [40]. Mann et al. [41] proposed a Duffing electromagnet oscillator that uses magnetic levitation to realize resonance tuning and bandwidth broadening. Chen et al. [42] modelled and fabricated a multi-degree of freedom EMG using vertical linear springs and nonlinear magnetic springs based on magnetic levitation. In addition to magnetic spring structure, a double-clamped beam structure with strong natural nonlinearity is also an effective method to broaden the frequency bandwidth. Lu et al. [43] proposed a nonlinear electromagnetic vibration energy harvester with a monostable double-clamp beam, and the average power and frequency bandwidth of the harvester reached 1.78 mW and 11 Hz, respectively, at 1 g acceleration.

Although the bandwidth of EMGs can be broadened in several ways, most of them use air as the magnetic induction line transfer medium between the magnet and the coil, which leads to weak magnetic coupling because of the low magnetic permeability of air. When soft magnetic materials with high permeability are introduced into electromagnetic energy harvesting applications, it leads to significant enhancement in output voltage and power density compared to energy harvesters based on air-cored coils [44]. Soft magnetic materials with high magnetic permeability can be used either as flexible cantilevers in oscillating structures [45] or as fixed elements in coils [46]. However, soft magnetic materials as oscillating structures may induce high stiffness, and in this case low resonant frequencies cannot be achieved. In contrast, placing the soft magnetic material statically in the coil can significantly increase the power density by the strong magnetic coupling between the fixed high-permeability soft magnetic material and the oscillating permanent magnet pair. Sun et al. [44] designed an EMG using a pair of antiparallel permanent magnets as the vibrating part and a soft magnetic material with high permeability as the static part in the coil, which combine to form a closed magnetic circuit and make the coupling force between the permanent magnets and the soft magnetic material nonlinear by adjusting the spacing. This EMG can simultaneously increase the output power and operating bandwidth.

In this work, a simple but effective approach to realize the nonlinear EMG with both wide bandwidth and high power based on magnetic coupling between the magnets and iron core is presented. A high-permeability soft magnetic material is placed in the center of the coil as a permeable core, and the strong nonlinear magnetic coupling between the oscillating magnet and the core enhances the magnetic flux inside the coil, thus increasing the output power. Its design, mathematical model, and simulation methods can be beneficial to the research of other EMGs, which is the contribution of this paper. The structure design and working principle are described and the equivalent dynamical model is developed in Section 2. Simulation and parameter optimization are provided in Section 3. Experimental setup and detailed tests are described in Section 4, together with discussion of test results and comparison to other low-frequency vibration generators reported in recent literatures.

## 2. Device Design and Theoretical Analysis

### Structural Design and Working Principle

**Figure 1:**
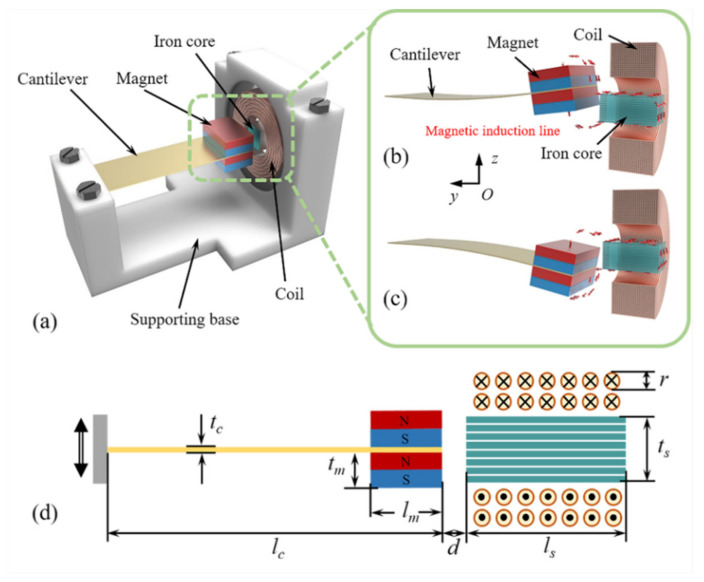
(**a**) Structure of the magnetic-coupled nonlinear electromagnetic generator (MNL-EMG). Distribution of magnetic induction lines around the magnets and iron core as the magnets are (**b**) above or (**c**) below the iron core. (**d**) Schematic diagram of the MNL-EMG.

The structure of the proposed magnetic-coupled nonlinear electromagnetic generator (MNL-EMG) is shown in Figure 1a. The MNL-EMG consists of a supporting base, a cantilever beam with two NdFeB magnets fixed at the free end, and a winding coil around a laminated iron core with high permeability. The material of the iron core is silicon lamination, which has 3% silicon content, which can increase the resistivity and maximum permeability of iron and reduce the core loss. The winding coil is wound with a diameter of 0.3 mm and a turn count of 500. The cantilever beam is made of phosphor bronze with good elasticity and non-magnetism. When the cantilever beam drives the end magnets to vibrate up and down, the iron core attracts the surrounding magnetic induction lines through the center of the coil. Therefore, the magnetic flux penetrating the coil changes, and an induced voltage will be generated across the coil. The magnetic force between the magnets and iron core varies nonlinearly with the displacement of the magnets. Hence the stiffness of the cantilever beam changes nonlinearly, resulting in the widening effect of operating bandwidth. Compared with the nonlinear EMGs relied on repulsion or attraction between magnets, this MNL-EMG based on the nonlinear attraction between the magnet and iron core has advantages of increasing the electromagnetic output power using a high-permeability iron core. Figure 1b,c shows the distribution of magnetic induction lines around the magnets and iron core when the magnets are above or below the iron core. The magnetic induction lines start from the N pole of the magnets, flow into the iron core, and then flow back to the S pole of the magnets. The iron core with high permeability can concentrate the magnetic induction lines inside the coil, enable the direction of magnetic induction lines perpendicular to the coil plane, greatly increases the magnetic flux, therefore increasing the induced voltage of the coil. The schematic diagram of the MNL-EMG is shown in Figure 1d, and the geometric parameters are listed in Table 1.

**Figure 2:**
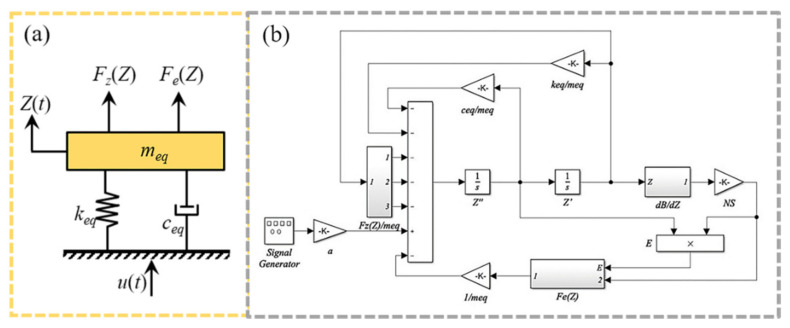
(**a**) Equivalent model and (**b**) MATLAB/Simulink diagram of the MNL-EMG.

An equivalent mass-spring-damper model of the MNL-EMG is developed as shown in Figure 2a. The corresponding dynamic equation can be expressed as:

(1) $$m_{eq}Z(t)+c_{eq}Z(t)+k_{eq}Z(t)=-m_{eq}u(t)-F_{z}(Z)-F_{e}(Z),$$

where *m_eq_*, *c_eq_*, and *k_eq_* represent the equivalent mass, equivalent damping, and equivalent stiffness of the cantilever beam, respectively; *Z* is the vertical displacement of the magnets at the free end of the cantilever beam; *u*(*t*) is the displacement of the external sinusoidal excitation; *F_z_* is the vertical component of the magnetic force *F_m_* between the magnets and iron core; and *F_e_* is the electromagnetic damping force between the magnets and coil. It is assumed that the displacement function *u*(*t*) of the sinusoidal excitation and the nonlinear magnetic force function *F_z_*(*Z*) are

(2) $$u(t)=\frac{a}{\omega^{2}}\sin(\omega t),$$

and

(3) $$F_{z}(Z)=p_{0}+p_{1}Z+\cdot \cdot \cdot \cdot \cdot \cdot +p_{n}Z^{n},$$

where *a* and *ω* are respectively the acceleration and angular frequency of the sinusoidal excitation; *p*_0_, *p*_1_⋯⋯*p_n_* are the coefficients of the polynomial function *F_z_*(*Z*).

The power generated by the electromagnetic damping force *F_e_* is equal to the product of the electromagnetic damping force and the relative movement speed, then the power is mainly consumed on the external load *R_load_* and the internal resistance *R_coil_* of the coil. Therefore, the instantaneous power *P_e_* can be expressed as

(4) $$P_{e}=F_{e}\frac{dZ}{dt}=\frac{E^{2}}{R_{coil}+R_{load}},$$

where *E* is the induced voltage of the coil, *dZ*/*dt* is the moving speed of the magnet. According to the law of electromagnetic induction, the induced voltage generated by the change of the magnetic flux in the closed coil can be expressed as [6]

(5) $$E=-\frac{d\Psi }{dt}=-N\frac{d\Phi }{dt},$$

where *Ψ* is the total magnetic flux, *N* is the turns of the coil, Φ is the magnetic flux passing through the single-turn coil, and Φ can be expressed as

(6) Φ=BScosθ,

where *B* is the magnetic flux density of each turn of the coil, *S* is the area enclosed by the coil, and *θ* is the angle between the normal vector of the coil plane and the direction of the magnetic field. It can be seen from Figure 1 that the angle between the normal vector of the coil plane and the direction of the magnetic field inside the iron core is equal to 0. By substituting Equations (5) and (6) into Equation (4), the electromagnetic damping force can be further expressed as

(7) $$F_{e}=NS\frac{dB}{dZ}\frac{E}{R_{coil}+R_{load}},$$

where *dB*/*dZ* is the gradient of the magnetic flux density along *z*-axis.

To calculate the electromechanical dynamics in a couple manner, this whole model with all above theories is established in MATLAB/Simulink, as shown in Figure 2b. The geometric dimensions used in the model are listed in Table 1.

## 3. Simulation Analysis and Optimization

### Analysis of Nonlinear Dynamics

**Figure 3:**
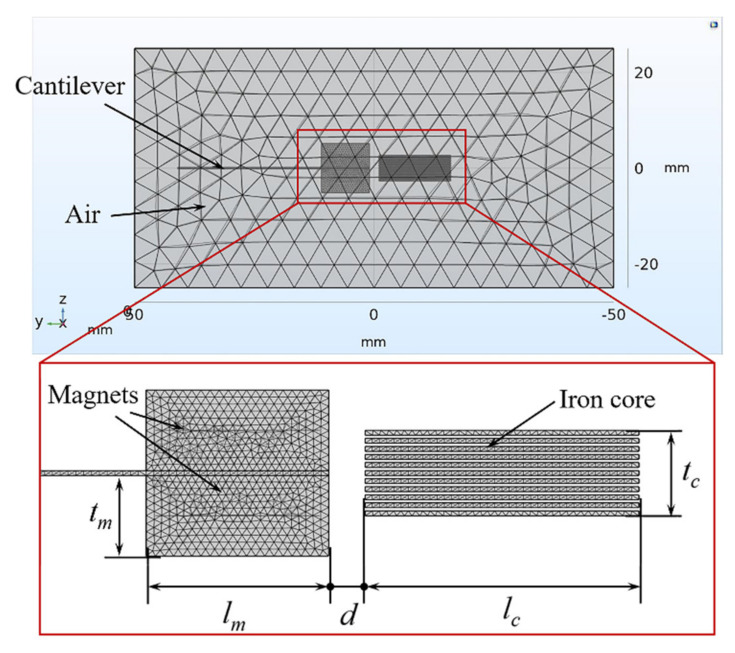
COMSOL magnetic field simulation model diagram.

**Figure 4:**
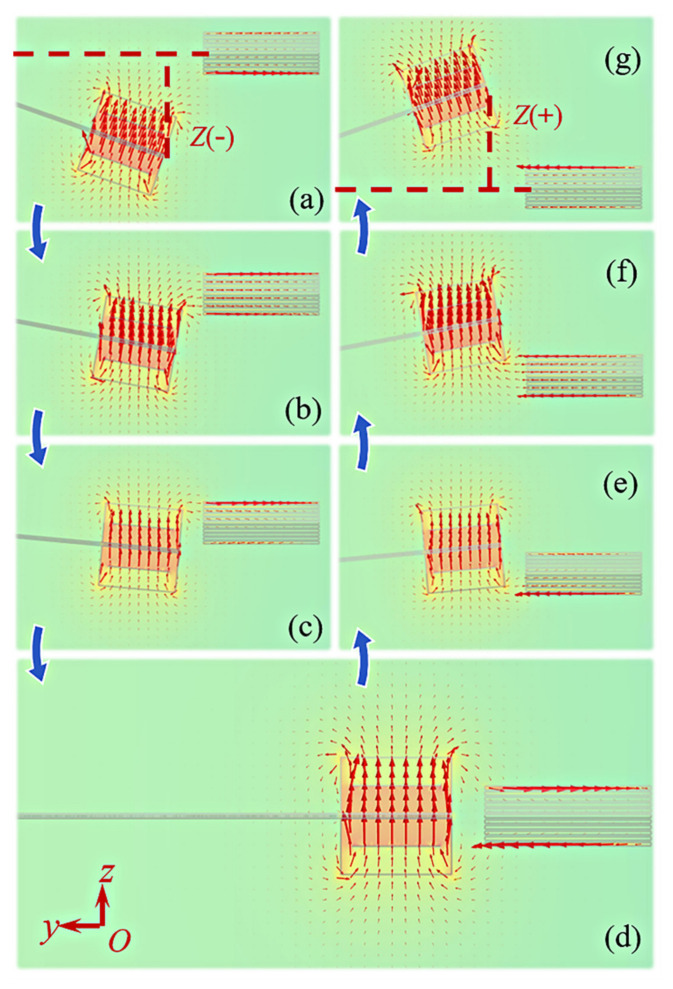
The magnetic field intensity and distribution at different displacements: (**a**) *Z* = 12 mm (point A); (**b**) *Z* = 8 mm (point B); (**c**) *Z* = 4 mm (point C); (**d**) *Z* = 0 (point D); (**e**) *Z* = −4 mm (point E); (**f**) *Z* = −8 mm (point F); (**g**) *Z* = −12 mm (point G).

Finite element analysis software COMSOL Multiphysics is used to simulate the magnetic field intensity and distribution at different displacements, and the simulation model is shown in Figure 3. The simulation results are shown in Figure 4. Placing an iron core near the magnets can guide the magnetic induction lines flowing from N pole of the magnets into the iron core, and then return to S pole after passing through the iron core. In addition to magnetic flux density inside the iron core, the magnetic force between the magnets and iron core can be also calculated by the magnetic field simulation. Using the curve fitting module in MATLAB, the polynomial function expression between the vertical magnetic force *F_z_* and the displacement *Z* is fitted as Equation (3), the polynomial function expression between the horizontal magnetic flux density *B_y_* and the displacement *Z* are fitted as

(8) $$B_{y}(Z)=q_{0}+q_{1}Z+\cdots \cdots +q_{m}Z^{m},$$

where *q*_0_, *q*_1_⋯⋯*q_m_* are the coefficients of the polynomial *B_y_*(*Z*). The *dB_y_*/*dZ* can be used to calculate the induced voltage of the coil.

**Figure 5:**
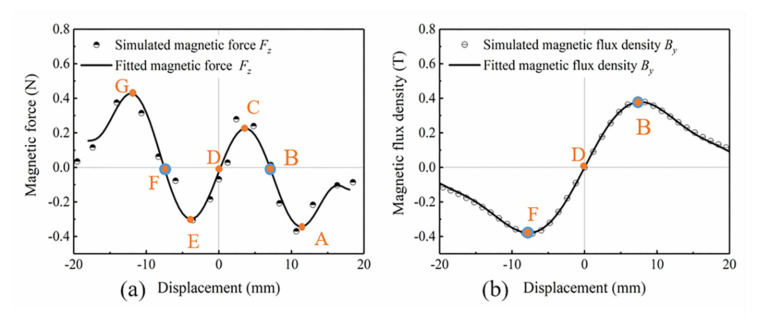
Fitted curves of (**a**) magnetic force *F_z_* and (**b**) magnetic flux density *B_y_* at distance of 2 mm.

Figure 5 shows the comparison of the simulated scatter points and the fitted curves at different displacements *Z* from −20 to 20 mm when the initial gap distance *d* between the magnets and iron core is 2 mm. Within a certain error range, the variation trend and numerical value of the simulated scatter points and the fitted curves are basically coincided. Figure 5a shows the fitted curve of the nonlinear magnetic force *F_z_*. When the value of *F_z_* is positive, it means that the magnetic force of the iron core to magnets is along the positive direction of *z*-axis. Conversely, when the value of *F_z_* is negative, the magnetic force of the iron core to magnet is along the negative direction of *z*-axis. As the displacement decreases from 20 mm to 0, the magnets are initially attracted by the iron core, and the attractive force gradually increases to the maximum value at displacement of 12 mm (point A). Then the attractive force drops to zero at displacement of 8 mm (point B). Next, the magnets are repelled by the iron core, and the repulsive force reaches the maximum value at 4 mm (point C), then decreases to zero at displacement of 0 (point D). Since the curve of *F_z_* is symmetrical about the origin of the coordinate axis, the magnets are repelled first and then attracted when the displacement decreases from 0 to −20 mm. The maximum repulsive force, the maximum attractive force, and the magnetic force equal to zero appear at −4 (point E), −12 (point F), and −8 mm (point G), respectively. After the magnetic force reaches the peak point G, the distance between the magnet and the iron core increases as the displacement increases, and the magnetic field strength around the magnet decreases, so the magnetic force decreases.

Correspondingly, the distribution of the magnetic field intensity and magnetic induction lines at point A to G is shown in Figure 4a–g, respectively, and the fitted curve of the magnetic flux density *B_y_* inside the iron core is depicted in Figure 5b. As shown in Figure 4a,g, the direction of magnetic induction lines inside the iron core at points A and G towards the positive and negative directions of *y*-axis, respectively. The iron core sheet closest to the magnet has the highest magnetic flux density, while the other sheets have very low magnetic flux density. The magnetic induction lines distribution at points B and F are shown in Figure 4b,f. It is seen that the magnetic induction lines introduced into the iron core are unidirectional and the number is the largest, so magnitude of *B_y_* at these two points is the highest, as shown in Figure 5b. At points C (Figure 4c) and E (Figure 4e), since the iron core is close to the N and S poles of the magnets, the magnetic induction lines inside the iron core are bidirectional, resulting in a decrease of *B_y_*. The magnetic induction lines start from the N pole into the iron core, then flow out from the iron core to the S pole. Figure 4d is the magnetic flux density distribution at point D. Here the N and S poles of the magnets are located on both sides of the iron core, the distance between the two magnetic poles and the center of the iron core is equal, so the magnetic induction lines flowing in and out of the iron core are equal. Thus, the magnetic flux density *B_y_* decreases to zero at point D.

**Figure 6:**
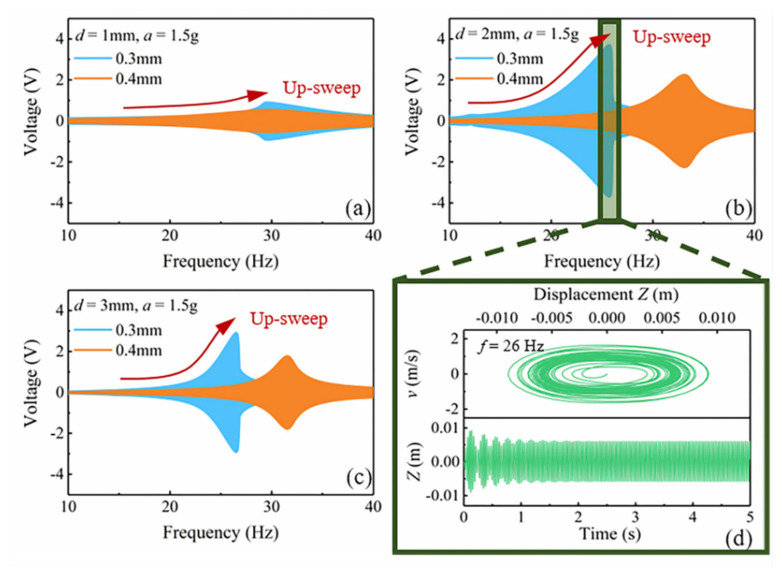
The simulated open-circuit voltage of the MNL-EMG with beam thickness of 0.3 mm and 0.4 mm, under gap distance of (**a**) 1 mm, (**b**) 2 mm, and (**c**) 3 mm. (**d**) The phase trajectory and displacement of the MNL-EMG with gap distance of 2 mm when the excitation acceleration *a* is 1.5 g and the excitation frequency *f* is 26 Hz.

Based on the COMSOL magnetic field simulation and MATLAB curve fitting, the functional expression of the nonlinear magnetic force *F_z_*(*Z*) and the gradient *dB_y_*/*dZ* of the magnetic flux density inside the iron core is obtained. According to the mathematical model, the voltage induced by the coil of the MNL-EMG under sinusoidal excitation is simulated. Since the gap distance between the magnets and iron core strongly affects the nonlinear magnetic force, the nonlinear response of the cantilever beam and the operating bandwidth of the MNL-EMG are simulated against different gap distances. Figure 6a–c depict the open-circuit voltage of the MNL-EMG with cantilever beam thickness of 0.3 and 0.4 mm against the frequency up-sweep as the gap distance *d* varying from 1, 2 to 3 mm, respectively, under acceleration of 1.5 g. It is seen that the MNL-EMG with beam thickness of 0.3 mm has higher voltage output and lower resonant frequency than that with beam thickness of 0.4 mm. The maximum output voltage occurs at gap distance of 2 mm and resonant frequency of 26 Hz. This is because the closer the magnets to the iron core, the greater the magnetic attraction along the *y*-axis direction. Once the gap distance is less than 1 mm, the magnet will be firmly attracted by the iron core, resulting in extremely low output voltage. As the gap distance increases, the attractive force along the *y*-axis direction decreases and the output voltage increases. However, the nonlinear magnetic force along the *z*-axis decreases with the increase of the distance. The nonlinearity of the voltage would be gradually weakened as the gap distance reaches 3 mm. The simulation results show that the MNL-EMG with a gap distance of 2 mm combines high output voltage and wide operating bandwidth. Figure 6d shows the phase trajectory and displacement of the MNL-EMG with gap distance of 2 mm when the excitation amplitude *a* is 1.5 g and the excitation frequency *f* is 26 Hz. The MB-EMG performs a large-amplitude periodic oscillation.

## 4. Experimental Results and Discussion

### 4.1. Experimental Setup

**Figure 7:**
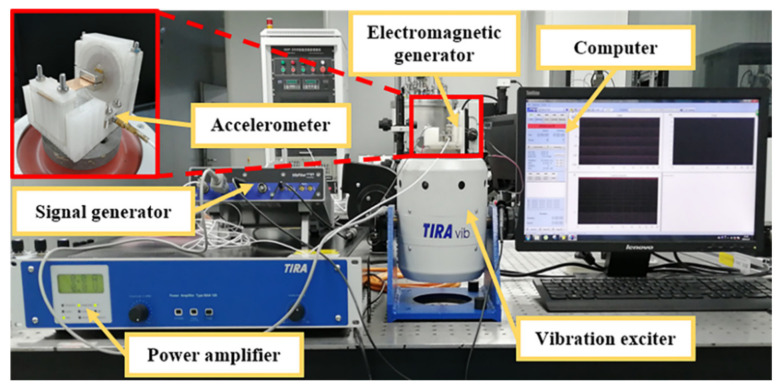
The experimental setup and the prototype.

The output performance of the MNL-EMG was tested by the vibration control and testing system as shown in Figure 7. The system setup consists of a computer, a vibration exciter (TIRA TV 511110, Germany), a power amplifier (BAA 120), a signal generator (Vipilot 4 channels) and an accelerometer (DYTRAN model 3035BG, USA). The MNL-EMG prototype is fixed on the vibration exciter, and the sinusoidal excitation of the vibration exciter is generated by the signal generator through the power amplifier. The accelerometer is installed on the exciter to monitor the excitation acceleration and realize the closed-loop feedback control of the vibration control system. The frequency and acceleration of the sinusoidal excitation can be adjusted by the control software in the computer. The voltage output of the MNL-EMG in frequency and time domains can be accurately measured and recorded by the dynamic signal analysis software (m+p international VibRunner).

### 4.2. Results and Discussion

**Figure 8:**
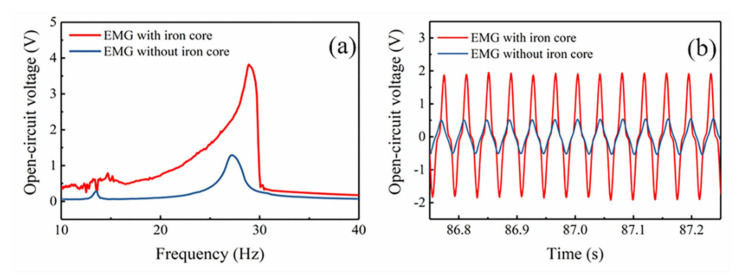
(**a**) Frequency domain and (**b**) time domain diagrams of the open-circuit voltage of the EMG with iron core and without iron core.

First, the EMG was subjected to a frequency upward sweep test using the vibration control and test system in the range of 10~40 Hz. As shown in Figure 8, the output performance of the EMG with and without iron core was compared under vibration acceleration of 1.5 g and gap distance of 2 mm. The resonant frequency of the EMG without iron core is 27 Hz, and the maximum output voltage at 27 Hz is 1.3 V. It is seen clearly that the voltage output of the MNL-EMG in frequency domain exhibits nonlinear characteristics, as shown in Figure 8a. The operating bandwidth of the MNL-EMG is 13 Hz (from 17 to 30 Hz), which is 4.3 times wider than that of the EMG without iron core, which is only 3 Hz (from 25.6 to 28.6 Hz). In addition, the high magnetic permeability of the iron core resulting in the increase of magnetic flux density inside the coil, so the output voltage of the MNL-EMG increases significantly. The maximum open-circuit voltage of the MNL-EMG (3.8 V at 29 Hz) is about 3 times higher than that of the EMG without iron core (1.3 V at 27 Hz). It is verified that the use of iron core with high magnetic permeability to the coil can broaden the operating bandwidth and improve the output performance effectively. The time domain diagrams of the open-circuit voltage of the EMG with iron core and without iron core are depicted in Figure 8b.

**Figure 9:**
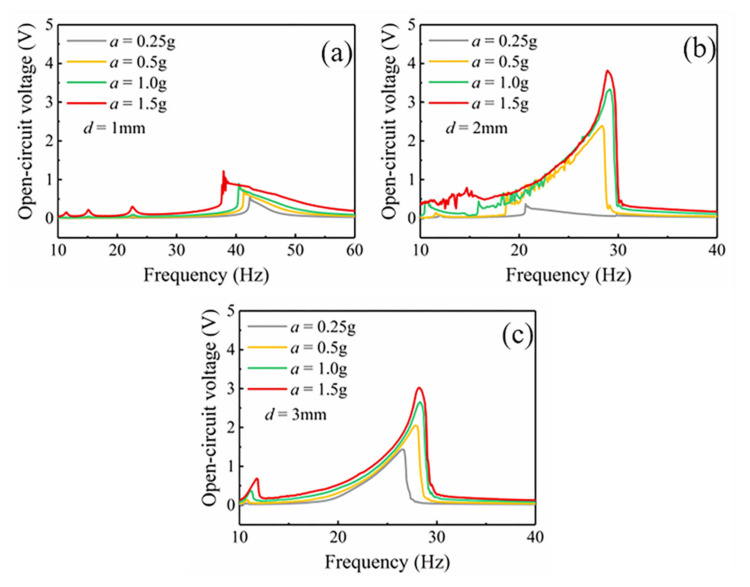
The open-circuit voltage outputs of the MNL-EMG at different accelerations under the distance *d* of (**a**) 1 mm, (**b**) 2 mm and (**c**) 3 mm, respectively.

**Figure 10:**
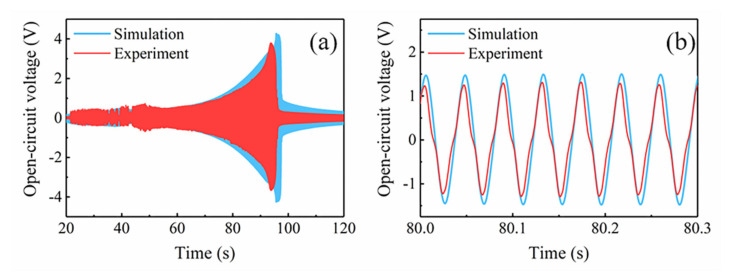
(**a**,**b**) The tested and simulated time domain waveform diagrams of the open-circuit voltage of the MNL-EMG at acceleration of 1.5 g, under distance of 2 mm.

The output performance of the MNL-EMG is strongly affected by the gap distance *d* between the magnet and iron core, as well as the acceleration of the excitation. Figure 9 shows the measured open-circuit voltage of the MNL-EMG against operating frequency at various acceleration levels of 0.25, 0.5, 1, and 1.5 g, under the gap distance of 1, 2, and 3 mm, respectively. It is seen the open-circuit voltage outputs of the MNL-EMG are quite low at the gap distance of 1 mm. This is because the magnetic attraction along *y*-axis is very strong, the deflection of the cantilever beam is strongly restricted by the magnetic attraction force. In contrast, under gap distance of 2 mm, the open-circuit voltage of the MNL-EMG is 2.39, 3.33 and 3.82 V at acceleration of 0.5, 1.0 and 1.5 g, respectively, which are higher than those of the MNL-EMG under gap distance of 3 mm. The maximum output voltage is 3.82 V at excitation frequency of 30 Hz. It should be noted that the open-circuit voltage of the MNL-EMG at acceleration of 0.25 g and under gap distance of 2 mm is 0.37 V, which is much lower than that of the MNL-EMG under gap distance of 3 mm. It can be inferred that greater acceleration is required to enhance the nonlinear response of the cantilever, therefore increasing the output voltage and broadening the bandwidth. Figure 10 shows the comparison of the simulated and tested waveform diagrams of the open-circuit voltage against frequency up-sweep at vibration acceleration of 1.5 g and under gap distance of 2 mm. The waveforms of the open-circuit voltage obtained by simulation and experiment are consistent, indicating that the established system dynamic model is suitable for this MNL-EMG device.

**Figure 11:**
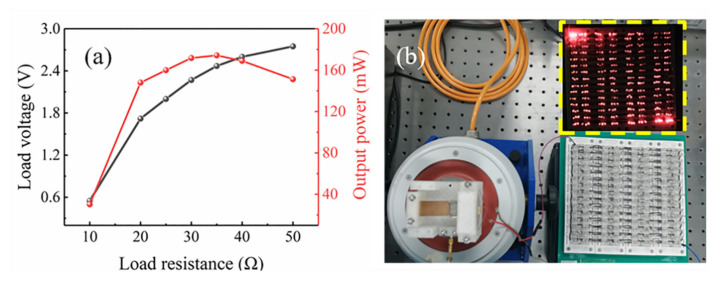
(**a**) The load voltage and maximum output power of the MNL-EMG versus load resistance. (**b**) Photograph of LEDs that are lighted up by MNL-EMG.

**Figure 12:**
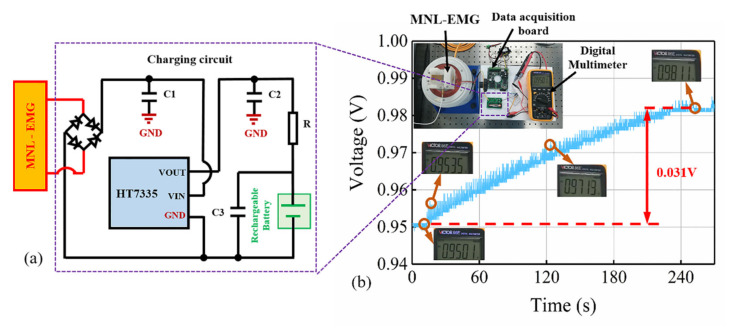
(**a**) Experimental circuit and (**b**) battery charging experiment.

Figure 11a shows the peak load voltage and maximum output power of the MNL-EMG versus load resistance at acceleration of 1.5 g and frequency of 30 Hz. The MNL-EMG can be regarded as a voltage source with an open-circuit voltage of *E* and an internal resistance of *r* equal to the resistance of the coil. With the increasing of load resistance, the load voltage continues to increase, while the corresponding power reaches to a maximum value of 174 mW at the optimal load resistance of 35 Ω. The maximum output power of MNL-EMG is 10.8 times higher than that of the EMG without iron core. Figure 11b demonstrated that the MNL-EMG can light up more than 360 parallel-connected LEDs. Moreover, to examine the charging performance of the MNL-EMG, the output of the MNL-EMG is connected to a rectifier to convert the AC input signal into a DC signal. Then the HT7335 voltage regulator chip converts the rectified DC voltage into a stable DC voltage to charge the battery, as shown in Figure 12a. The rechargeable battery is a Ni-MH battery with 1.2 V nominal voltage and 900 mAh nominal capacity. Figure 12b presents the voltage of the battery during the experiment. The battery voltage was 0.95 V at the beginning. After the charging process of 240 s, the stable voltage of the rechargeable battery increased to 0.98 V. In real applications, the MNL-EMG is designed to harvest the vibration energy generated by these mechanical devices, such as vibrating screen, coal mining machine, pump, gear box, etc., to power the sensors and eventually build a self-powered wireless sensing network.

Performance comparison between the introduced MNL-EMG and recent published low-frequency vibration generators in the literature is given in Table 2. It is worth pointing out that a fair comparison may need to re-design the energy harvester for the specific working condition, which is quite difficult. The acceleration, operating frequency range, and power density somehow generalized working condition and performance parameter. As the data shown in Table 2, previous works investigated different transaction mechanisms for the low-frequency vibration. The MNL-EMG in this work can generate a high power output and a wide bandwidth at relatively large acceleration, which indicates its superior performance over other vibration generators in the large-amplitude vibration environment. In general, the introduced MNL-EMG device has achieved high output power, wide working bandwidth, and low working frequency, simultaneously. It can easily capture the low-frequency environmental vibration energy, and the output power is sufficient to power micro electronic devices. In the subsequent research, we will continue the integration of the power management circuit for the MNL-EMG and build a complete power supply system, which would have broad application prospects.

## 5. Conclusions

This paper presents a MNL-EMG with wide bandwidth and high performance. In this MNL-EMG, advantages of several technologies are combined to enhance the output performance: (1) the nonlinear attraction between magnets and iron core enable the cantilever beam to operate in a high-energy orbit to generate wider operating bandwidth and higher output power; (2) iron core with high-permeability guides the magnetic induction lines into the coil, therefore greatly increased the electromagnetic output power while performing nonlinear motion. To reveal the mechanism of nonlinear effect, the magnetic force of the iron core to the magnets and the magnetic flux density inside the iron core at different displacements are simulated and analyzed. Based on the dynamic model, the output voltage of the MNL-EMG is simulated, and the dimension parameters are optimized. Then a series of experiments are conducted to test the output performance of the prototype. Under an acceleration of 1.5 g, the MNL-EMG can maintain high output power in the ultra-wide frequency range of 17~30 Hz, and the operating bandwidth reaches 13 Hz, which is 4.3 times wider than that of linear EMG without an iron core. The maximum output power of the MNL-EMG reaches 174 mW under the optimal load resistance of 35 Ω, and the prototype can light up 360 LEDs. Moreover, the prototype can charge a Ni-MH rechargeable battery (1.2 V, 900 mAh) from 0.95 V to 0.98 V within 240 s. It is proved that the proposed MNL-EMG has advantages of wide operating bandwidth and extremely high output power. This research can be of great significance for further exploration of nonlinear vibration energy harvester.

## Figures and Tables

**Table 1:** Geometric dimensions of the MNL-EMG.

Parameter	Value
Length of the cantilever beam *l_c_*	40 mm
Width of the cantilever beam *W_c_*	13 mm
Thickness of the cantilever beam *t_c_*	0.3 mm
Length of the magnet *l_m_*	10 mm
Width of the magnet *W_m_*	15 mm
Thickness of the magnet *t_m_*	5 mm
Length of the iron core *l_s_*	15 mm
Width of the iron core *W_s_*	7 mm
Thickness of the iron core *t_s_*	7 mm
Distance between the magnet and iron core *d*	2 mm
Diameter of the copper wire *r*	0.3 mm
Diameter of the coil *R*	30 mm
Turns of the coil *N*	500

**Table 2:** Performance comparison between the MNL-EMG and low-frequency vibration generators reported in the literature.

Reference	Transducer	Acceleration (g)	Frequency (Hz)	Bandwidth (Hz)	Power (mW)
Ren [47]	PE	0.2	12~14.5	2.5	1.40
Shen [6]	EM	3.25	3~6	3	23.2
Yan [7]	EM	0.6	5~15	10	28
Aldawood [40]	EM	0.4	7~12	5	80
Sun [44]	EM	0.5	57.4~64.7	7.3	0.003
Haroun [48]	EM	1.26	3.33	--	0.083
Gu [49]	EM	1.0	5~27	22	7.65
Toyabur [27]	EM and PE	0.4	12~22	10	0.49
Fan [50]	EM and PE	1.5	6~8.5	2.5	1.42
Salauddin [51]	EM and TE	0.5	5	--	11.75
Askari [52]	EM and TE	0.57	45.45~55.45	10	74
This work	EM	1.5	17~30	13	174

g = 9.8 m/s^2^, PE = piezoelectric, EM = electromagnetic, TE = triboelectric.
